# THE ROLE OF SOCIAL DISCONNECTION AND THE NEIGHBORHOOD EXPOSOME IN COGNITIVE DECLINE AMONG BLACK AMERICANS

**DOI:** 10.1093/geroni/igae098.1171

**Published:** 2024-12-31

**Authors:** Ann Nguyen, Weidi Qin, Harry Taylor

**Affiliations:** Case Western Reserve University, Cleveland, Ohio, United States; University of Wisconsin Madison, Madison, Wisconsin, United States; University of Toronto, Toronto, Ontario, Canada

## Abstract

The aims of this study were to 1) identify cognitive status trajectories among middle-aged and older Black adults; 2) determine whether baseline loneliness, social isolation, and neighborhood characteristics predicted cognitive status trajectories; and 3) determine whether neighborhood characteristics modified the association between loneliness, social isolation, and cognitive status trajectories. Data for the study came from Black participants aged 51+ in the Health and Retirement Study (N=1791). Cognitive status was measured using the modified Telephone Interview for Cognitive Status; loneliness was measured using the modified UCLA Loneliness Scale. Social isolation from extended family, children, and friends were measured using indicators of network size, contact, and social support for each social group. Neighborhood physical disadvantage and social cohesion were each assessed using a 4-item scale. Group-based trajectory analysis was used. The results indicated that there were four distinct cognitive status trajectories—very low and declining, low and declining, moderate and stable, and high and stable. Higher loneliness scores were predictive of membership in the low and declining trajectory rather than the high and stable trajectory. A significant interaction between neighborhood physical disadvantage and loneliness indicated that the association between loneliness and membership in the low and declining trajectory was weaker among participants who lived in neighborhoods with high levels of physical disadvantage than participant who lived in neighborhoods with low levels of physical disadvantage. These findings have implications for practice with older Black adults and suggest that practitioners should consider the role of loneliness and the neighborhood environment.

